# Infant and childhood neurodevelopmental outcomes following prenatal exposure to selective serotonin reuptake inhibitors: overview and design of a Finnish Register-Based Study (FinESSI)

**DOI:** 10.1186/1471-244X-12-217

**Published:** 2012-12-04

**Authors:** Heli Malm, Miia Artama, Alan S Brown, Mika Gissler, David Gyllenberg, Susanna Hinkka-Yli-Salomäki, Ian McKeague, Andre Sourander

**Affiliations:** 1 Teratology Information, HUSLAB and Helsinki University Central Hospital, Tukholmankatu 17, P.O. BOX 790, 00029 HUS, Helsinki, Finland; 2 Department of Clinical Pharmacology, Helsinki University and Helsinki University Central Hospital, Helsinki, Finland; 3 Department of Child Psychiatry, University of Turku, Turku, Finland; 4 National Institute for Health and Welfare, Helsinki, Finland; 5 Department of Psychiatry, Columbia University College of Physicians and Surgeons, New York State Psychiatric Institute, New York, NY, USA; 6 Department of Epidemiology, Columbia University, Mailman School of Public Health, New York, NY, USA; 7 Nordic School of Public Health, Gothenburg, Sweden; 8 Department of Child Psychiatry, University of Helsinki, Helsinki, Finland; 9 Mailman School of Public Health, Department of Biostatistics, Columbia University College of Physicians and Surgeons, New York, NY, USA

**Keywords:** SSRI, Pregnancy, Neurodevelopment

## Abstract

**Background:**

Experimental animal studies and one population-based study have suggested an increased risk for adverse neurodevelopmental outcome after prenatal exposure to SSRIs. We describe the methods and design of a population-based study examining the association between prenatal SSRI exposure and neurodevelopment until age 14.

**Methods and design:**

This is a cohort study of national registers in Finland: the Medical Birth Register, the Register of Congenital Malformations, the Hospital Discharge Register including inpatient and outpatient data, the Drug Reimbursement Register, and the Population Register. The total study population includes 845,345 women and their live-born, singleton offspring aged 14 or younger and born during Jan 1^st^ 1996-Dec 31^st^ 2010. We will compare the prevalence of psychiatric and neurodevelopmental outcomes in offspring exposed prenatally to SSRIs to offspring exposed to prenatal depression and unexposed to SSRIs. Associations between exposure and outcome are assessed by statistical methods including specific modeling to account for correlated outcomes within families and differences in duration of follow-up between the exposure groups. *Descriptive results.* Of all pregnant women with pregnancy ending in delivery (n = 859,359), 1.9% used SSRIs. The prevalence of diagnosed depression and depression-related psychiatric disorders within one year before or during pregnancy was 1.7%. The cumulative incidence of registered psychiatric or neurodevelopmental disorders was 6.9% in 2010 among all offspring born during the study period (age range 0–14 years).

**Discussion:**

The study has the potential for significant public health importance in providing information on prenatal exposure to SSRIs and long-term neurodevelopment.

## Background

Safety of the selective serotonin reuptake inhibitors (SSRIs) during pregnancy is a question of major clinical importance as up to 6% of pregnant women are reported to use SSRIs [1]. Every sixth pregnant woman suffers from major depressive disorder, and while untreated prenatal depression can have devastating consequences for the mother and her family, it has also been linked to poor perinatal outcome [2,3]. SSRIs are not major teratogens but neonatal adaptation problems are common after prenatal exposure [4-7] and an increased risk has been observed for other perinatal complications including persistent pulmonary hypertension [8-10]. Consequently, both patients and clinicians frequently need to balance between the risks of untreated depression and the potential risks associated with continuing antidepressant treatment during pregnancy.

Serotonin has a crucial role in neural development and maturation [11] and studies in rodents have demonstrated that manipulation of serotonin (5-HT) levels by exposing the animals to SSRIs at a vulnerable stage of central nervous system development can lead to permanent behavioral changes, paradoxically presented as increased depression and anxiety-related behavioral phenotypes [12-15].

In humans, long-term effects of prenatal SSRI exposure on childhood neurodevelopment have not been extensively studied. While small, non-population based cohort studies observed no excess risk by age four or seven [16-18], one population-based study suggested an increased risk of autism spectrum disorders in prenatally exposed offspring [19]. A major challenge remains to disentangle the effects of maternal depression and maternal use of SSRIs on later neurodevelopment in offspring. This study represents the epidemiological part of an international, collaborative research project which is part of a Conte Center project for the Neuroscience of Mental Disorders at Columbia University (http://columbiapsychiatry.org). This innovative collaboration consists of several linked research projects addressing the fundamental questions of altered serotonin signalling and prenatal SSRI exposure on brain structure, function, and long-term behavioural outcomes. Other projects in this collaboration include basic and experimental research with animal models, and clinical neurobiological studies on infant/childhood outcomes following prenatal SSRI exposure*.* These studies will help to interpret and validate our findings derived from epidemiologic research, while our findings may help generate novel hypotheses to be tested in animal models. This concept of translating basic research findings to epidemiology and *vice versa* constitutes a model of bidirectional translational epidemiology [20]. Because of the steadily increasing use of SSRIs during pregnancy, this is one of the most challenging questions in prenatal psychiatric epidemiology at present and carries significant public health importance.

The objective of the nationwide **Fin**nish Register-Based Study on Infant and Childhood Neurodevelopmental Outcomes Following Prenatal **E**xposure to **S**elective **S**erotonin Reuptake **I**nhibitors (FinESSI) is to investigate if prenatal SSRI exposure increases the risk of adverse psychiatric or neurodevelopmental outcome until age 14, controlling for maternal depression. The aim of this methods paper is to describe the study design, the national registers included in the study, and linkage of the registers. We also report the trends in SSRI use during pregnancy during the study period, the prevalence of diagnosed depression and depression-related psychiatric disorders in the pregnant population, and the cumulative incidence of psychiatric and neurodevelopmental disorders in the whole offspring population.

## Methods and design

### Overview of the study design

**Figure 1:**
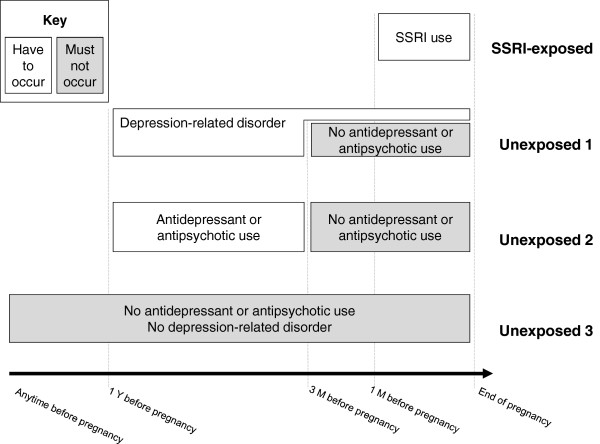
**Time-window criteria for definition of exposed and unexposed offspring.** SSRI, selective serotonin reuptake inhibitor; Y, year; M, months. *SSRI exposed:* Offspring exposed prenatally to SSRIs. *Unexposed 1*: All offspring unexposed prenatally to antidepressants and antipsychotics, but mother treated for depression or depression-related disorder during pregnancy or within one year before pregnancy. *Unexposed 2*: Offspring unexposed prenatally to antidepressants and antipsychotics but mother has used antidepressants or antipsychotics within one year before pregnancy. *Unexposed 3*: Offspring unexposed prenatally to antidepressants and antipsychotics and mother has no history of psychiatric treatment (inpatient or outpatient diagnosis or use of antidepressants or antipsychotics).

This is a population-based, prospective cohort investigation. All data are collected from national, population-based registers. Psychiatric and neurodevelopmental diagnoses in offspring exposed prenatally to SSRIs are compared to unexposed offspring controlling for maternal depression (Figure 1). The sampling frame includes 845,345 offspring consisting of all singleton live births in Finland (approximately 56,500 births per year) between Jan 1^st^ 1996 and Dec 31^st^ 2010, identified from the Medical Birth Register. Hence, the age range of children born in this cohort will be from birth to age 14. Data on SSRI purchases are collected from the Drug Reimbursement Register. The follow-up for psychiatric and neurodevelopmental disorders is based on collection of these diagnoses from the Hospital Discharge Register. Data on major congenital anomalies are collected from the Register of Congenital Malformations, and parental background data from the Population Register. Detailed description of the methods follows.

### Description of the registers

*The national Drug Reimbursement Register (DRR),* maintained by the Social Insurance Institution in Finland (KELA) since 1995, tracks drugs that have been purchased from pharmacies and contains data on 99% of reimbursed prescription drug purchases [21]. Prescription-only medicines deemed necessary for the treatment of an illness are reimbursed under the Social Insurance System which covers all permanent residents in Finland. Drug purchases are reimbursed concomitantly upon purchase at pharmacies and drugs are supplied to the patient for a maximum of three months at a time. Data in the register include the date of the purchase, the International Anatomic-Therapeutic-Chemical (ATC) classification code indicating the generic name of the drug and the dose prescribed. KELA also maintains *the Special Reimbursement Register* since 1964 with data on several chronic illnesses requiring continuous drug treatment, with available information on possible special reimbursement status including indication for treatment. Over-the-counter drugs or medications given to institutionalized persons are not included in the register.

*The national Medical Birth Register (MBR)* was established in 1987 and is maintained by the National Institute for Health and Welfare (THL). This computerized registry collects data on maternal demographic characteristics, medical history including reproductive history, health-related behaviours, diagnoses during pregnancy and delivery, and neonatal outcome data up to seven days’ age. Data in the MBR are collected in a standard form from all maternity hospitals and include all births, including the occasional homebirths. All infants are examined in the hospital by a paediatrician. All live births and stillbirths with gestational age of 22 weeks or more or birth weight of 500 grams or more are included in the register. The register data are confirmed and complemented from the maternity hospital records in cases of conflicting or missing information. The definitions and variables included in this registry are based on established international concepts and use the 10^th^ version of the WHO International Classification of Diseases (ICD). Extensive review of the data, including cross-checking with the data from the Finnish Population Register and Cause-of-Death Register at Statistics Finland indicate that the data are virtually complete [22,23].

*The national Hospital Discharge Register (HDR)* contains a hospital identification code and data on admission and discharge dates as well as primary and secondary diagnoses (two for inpatient care and 20 for outpatient care) at discharge. The register covers all hospital inpatient episodes in public and private institutions and outpatient hospital visits in public hospitals. The diagnoses are coded using ICD-8 (1969–1986), ICD-9 (1987–1995), and ICD-10 since 1996. Data on hospital discharges are available from 1969, and data on all contacts in outpatient clinics are available from 1998 until the present. These registries will be used to identify the recorded diagnoses for all psychiatric hospital admissions since 1969 for parents and grandparents, as well as hospital admission care (since birth) and outpatient psychiatric contacts (since birth, starting from 1998) for the child. The HDR data have been validated for psychiatric diagnoses and are considered good [24-26]. In Finland, all children attend regularly child welfare clinics where medical examinations are performed by a trained public health nurse or a physician, and which are mandatory and free of charge. Data from these clinics are entered into the hospital and outpatient registries if the child is referred for specialized care for identified conditions. These services are particularly designed to identify psychiatric and neurodevelopmental disorders. Annual examinations are further performed for all school children and when indicated, a referral is made to special health care. All diagnoses made by these health care providers are recorded in the inpatient and outpatient registries.

*The national Register of Congenital Malformations (RCM),* maintained by THL, receives comprehensive data on congenital anomalies including live births, stillbirths and fetuses from pregnancy terminations due to severe fetal anomaly, all with at least one detected major congenital anomaly including major structural anomalies, chromosomal defects and congenital hypothyroidism, classified and coded according to the 9^th^ version of the ICD classification. Minor anomalies are excluded principally according to the exclusion list of the European Surveillance of Congenital Anomalies, EUROCAT [27]. Data are collected from hospitals, health-care professionals and cytogenetic laboratories. The RCM also draws upon data from the Medical Birth Register and other relevant health registers. The diagnoses obtained from these data sources are confirmed by systematic contacts with hospitals that have given treatment to the infant. Although the register mainly collects data from the first year of the infant’s life, it also includes subsequently detected congenital anomalies. The validity of the RCM is considered good and has been ascertained in several studies [28-30].

*The national Population Register (PR*), maintained by the Finnish Population Register Centre is a computerized register that contains basic information, including country of birth, marital status, marriages and divorces, and deaths of all Finnish citizens and other citizens residing permanently in Finland.

### Exposure groups

The beginning of pregnancy has been calculated from the best clinical estimation of gestational age at birth (primarily based on ultrasound) as registered in the MBR. SSRI drug purchase(s) (including fluoxetine, citalopram, paroxetine, sertraline, fluvoxamine and escitalopram) during the period from one month before pregnancy until the end of pregnancy indicate exposure and the date of drug purchase is an indicator for beginning of exposure. Length of exposure is defined by using an algorithm based on data on the Defined Daily Dose (DDD) obtained from the DRR. A sub-analysis to evaluate overall SSRI exposure and exposure to individual SSRIs during different trimesters is based on dividing the trimesters into first (days 0–84 after the beginning of pregnancy), second (days 85–182), and third trimester (days 183 after the beginning of pregnancy until birth). The total number of exposed children included in this cohort is appr. 15,000.

The unexposed comparison groups are described below and in Figure 1. The object of the first two comparison groups is to control for maternal depression.

The unexposed group 1 includes all offspring of women with no purchases of antidepressants (ATC codes N06A, N06CA) or antipsychotics (N05A) from three months before pregnancy until the end of pregnancy, but with a diagnosis of depression or other psychiatric disorder related to depression or SSRI use during the period from one year before until the end of the index pregnancy (Figure 1). The diagnostic inclusion criteria are schizophrenia, schizotypal and delusional disorders (ICD-10 F20-F29), mood disorders (F30-F39), and neurotic, stress-related and somatoform disorders (F40-F48). Within the total pregnant population, 14,506 (1.7%) of women met the inclusion diagnosis (National Institute for Health and Welfare, unpublished statistics, Finland 2012). We estimate that more than half of these women have used antipsychotics or antidepressants within three months before pregnancy until the end of pregnancy, leaving 5,000 to 6,000 offspring to be included in this reference group.

The unexposed group 2 includes offspring of women with antidepressant or antipsychotic drug purchases during the period from 1 year until 3 months prior to pregnancy but no antidepressant or antipsychotic drug purchases during the period of 3 months prior to pregnancy until end of pregnancy, excluding offspring included in the first comparison group (Figure 1). The use of SSRIs has been steadily increasing and at present, 10% of women of child-bearing age use antidepressants in Finland, a figure which is nearly three times more common than during pregnancy (National Institute for Health and Welfare, unpublished statistics, Finland 2012). Accordingly, a total of 40,000 women (4-5%) of the study population are expected to have used antidepressant medication prior to pregnancy. By subtracting the SSRI -exposed group (n = 15,000) and the ‘unexposed 1’ group (n = 5,000) from these 40,000 women, we expect to obtain a total of 20,000 offspring in this group. However, the numbers expected to be obtained in ‘unexposed 1’ and ‘unexposed 2’ groups are based on an estimate and may differ from the final numbers. Due to the relatively small expected numbers in these groups no matching will be performed.

The unexposed group 3 serves as a ‘healthy’ background group and consists of offspring of pregnant women with no purchases of antidepressants or antipsychotics at any time prior to pregnancy or during pregnancy. In this group, the mother has neither diagnosis of depression or a psychiatric disorder related to depression, nor SSRI use at any time prior to pregnancy or during pregnancy. Two (2) unexposed per one (1) exposed are selected randomly from a cohort matched for date of birth within a period of +/−6 months. Such matching will allow similar follow-up periods between exposed and unexposed offspring. The total number of offspring in this group will be appr. 30,000.

### Definition of outcomes

We will focus on infant/childhood/diagnosed psychiatric and neurodevelopmental outcomes defined by ICD-10 codes under chapter on Mental and behavioral disorders’ (F00-F99), obtained from the HDR and including e.g. depression (F32-39), anxiety disorders (F40-42), mental retardation (F70-79), learning disabilities and abnormalities in motor development (F80-83), autism spectrum disorders (F84), attention deficit hyperactivity disorder (F90), and oppositional and conduct disorders (F91-92).

*Covariates and possible confounders* to be considered for adjustment in the analyses are presented in Table 1. These data are virtually complete apart from data on 5 minute Apgar score which are only available from the beginning of year 2004, and data on smoking which are missing in 3% of the study material.

**Table 1:** Covariates and possible confounders to be considered for adjustment in the analyses

**Mother**	**Age**
	Marital status
	Parity
	Smoking
	SES
	Depression or depression-related psychiatric diagnosis after the index pregnancy
	Exposure to other psychiatric drugs or suspected or established teratogens
Pregnancy	Pregnancy-related diagnoses and complications
	Mode of delivery
Perinatal events	Preterm birth (<37 weeks)
	Low 1 and 5 -minute Apgar score (<7)
	Fetal hypoxia (pH < 7.25)
	Low birth weight (<2.500 g)
	Small for gestational age
	Major congenital anomaly
Family	Parental death, divorce and country of birth
	Paternal and grandparents’ psychiatric diagnoses

SES, socio-economic status.

### Data linkages

**Figure 2:**
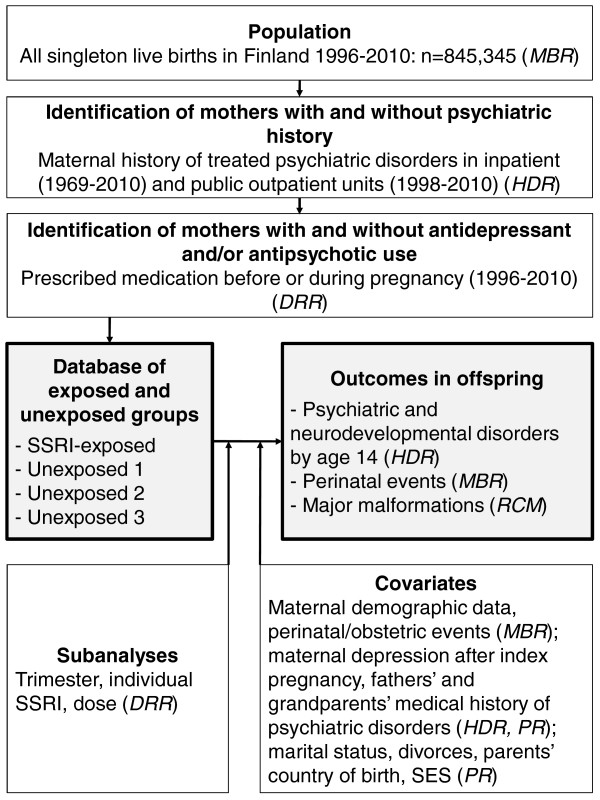
**Flow chart of the register-based sources of information used in the study.** MBR, Medical Birth Register; RCM, Register of Congenital Malformations; DRR, Drug Reimbursement Register; HDR, Hospital Discharge Register; SSRI, selective serotonin reuptake inhibitor; PR, Population Register; SES, socio-economic status.

All linkages are made by the unique personal identity code (PIC) which was introduced in 1964–1968 and is assigned to all Finnish citizens and permanent residents in Finland. The PIC is obligatory in every register and remains unchanged throughout a person’s lifetime. First, offspring from the total population, exposed and unexposed to SSRIs *in utero* are identified using linkages between the MBR and the DRR (drug reimbursement data) (Figure 2). Second, these data are linked to the HDR, and maternal exposure to depression and depression-related disorders is defined according to information on maternal psychiatric diagnosis in this register. Third, based on the criteria described previously (Figure 1) and the linkage procedure described in Figure 2, the exposed-unexposed database is formed. Fourth, pregnancy and infant data from the MBR and data on infant and childhood psychiatric and neurodevelopmental disorders from the HDR, and data on covariates are added to the database. Identification of biological fathers and grandparents and information on parental background data are obtained from the PR. The database is further expanded to include information on paternal and grandparents’ psychiatric diagnoses from the HDR, and information on perinatal and infant health outcomes from the MBR and the RCM.

The utilization of sensitive health register data for scientific research and the data linkages has been approved by the register administrators and the data protection authority. The study protocol was approved by the Institutional Ethical Review Board at THL and by the Institutional Review Board of the New York State Psychiatric Institute. Since the study subjects were not contacted, informed consent was not required.

### Statistical analyses

All data are anonymized and coded prior to statistical analysis. The incidence of specific offspring outcomes are compared between exposed and unexposed offspring. Univariate analyses are used to study demographic differences between the study cohorts, and logistic regression to assess the association between prenatal SSRI exposure and infant/childhood neurodevelopmental outcomes. Random-intercept logistic regression models are used to account for correlated outcomes within families (siblings), and Cox proportional hazards regression analyses to study potential differences in duration of follow-up between exposed and unexposed cohorts. In addition, we will utilize a class of multivariate failure time frailty models [31] to assess the effect of SSRI exposure on the specified outcomes, with subject-specific gamma frailties accounting for the correlation in the times to diagnosis of the different outcomes. Further analyses will focus on differences in outcome variables according to trimester of exposure and on individual drug level considering drug dose.

### Statistical power

With 15,000 exposed infants, the study has 80% power to detect a 1.3-fold increase in prevalence of perinatal outcomes and psychiatric disorders that have a prevalence of 1% in the entire cohort (alpha = 0.05, two-sided); for disorders that have prevalences of 3% and 5%, we have 80% power to detect 1.2-fold and 1.1-fold increases in prevalence, respectively.

#### Descriptive results

**Figure 3:**
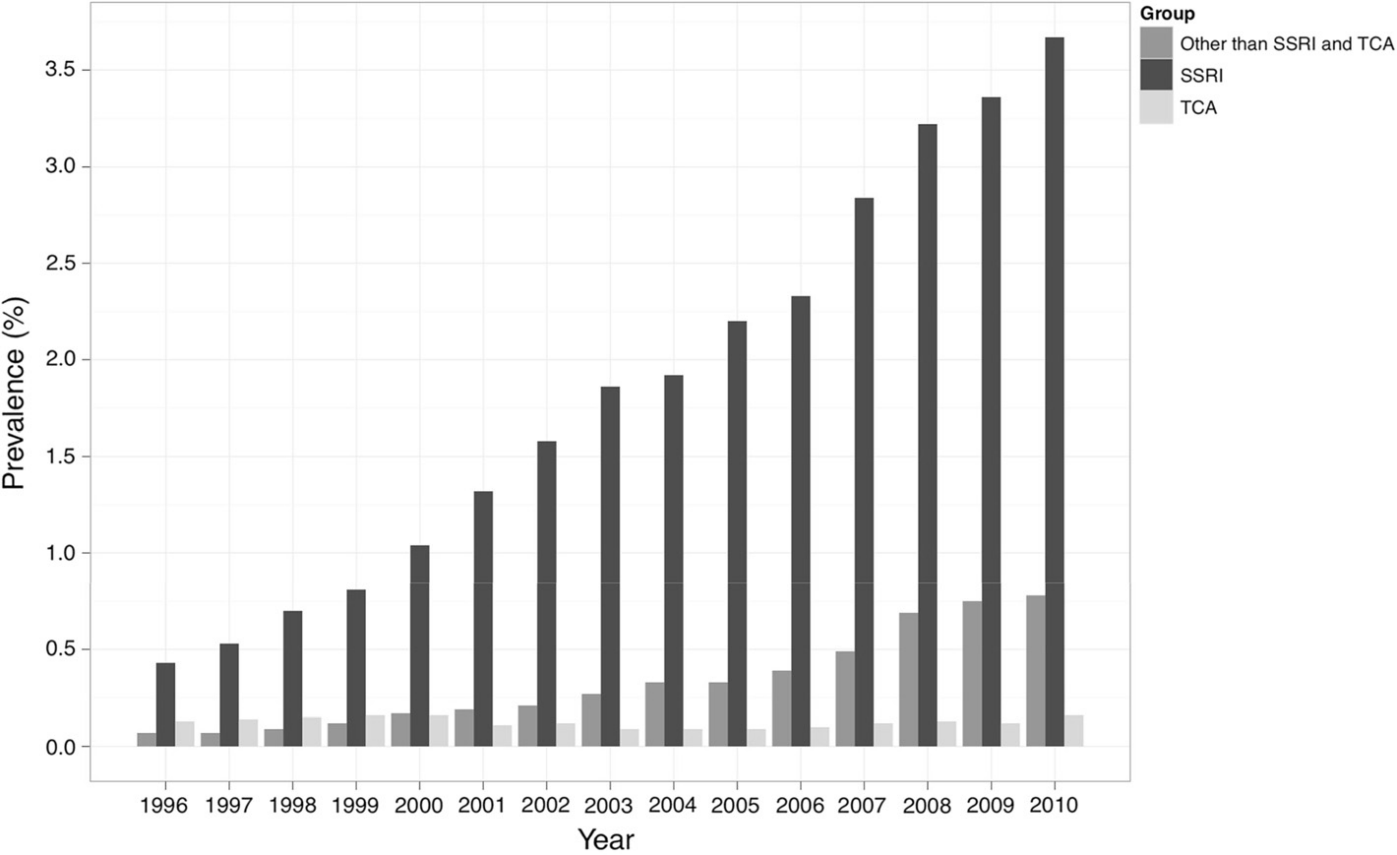
**Time-trend in antidepressant use among pregnant women in Finland, years 1996–2010.** The numbers include antidepressant drug purchases from one month before until the end of pregnancy. SSRI, selective serotonin reuptake inhibitor; TCA, tricyclic antidepressant.

The use of antidepressants within the whole pregnant population and including all pregnancies ending in delivery (n = 859,359) increased steadily during the study period from 0.6% in 1996 to 4.8% in 2010. This increase was largely due to the increasing use of SSRIs, which were used by 0.4% of pregnant women in 1996, and 3.8% in 2010, while the use of tricyclic antidepressants remained largely unchanged (Figure 3). The most commonly used SSRI was citalopram (0.8% of pregnant women), followed by fluoxetine (0.4%) and sertraline (0.3%). During pregnancy, SSRIs were most commonly purchased during the first trimester or one month before pregnancy (1.6%), with a declining prevalence as pregnancy advanced (0.8% of pregnant women in the second trimester and 0.7% in the third trimester).

**Figure 4:**
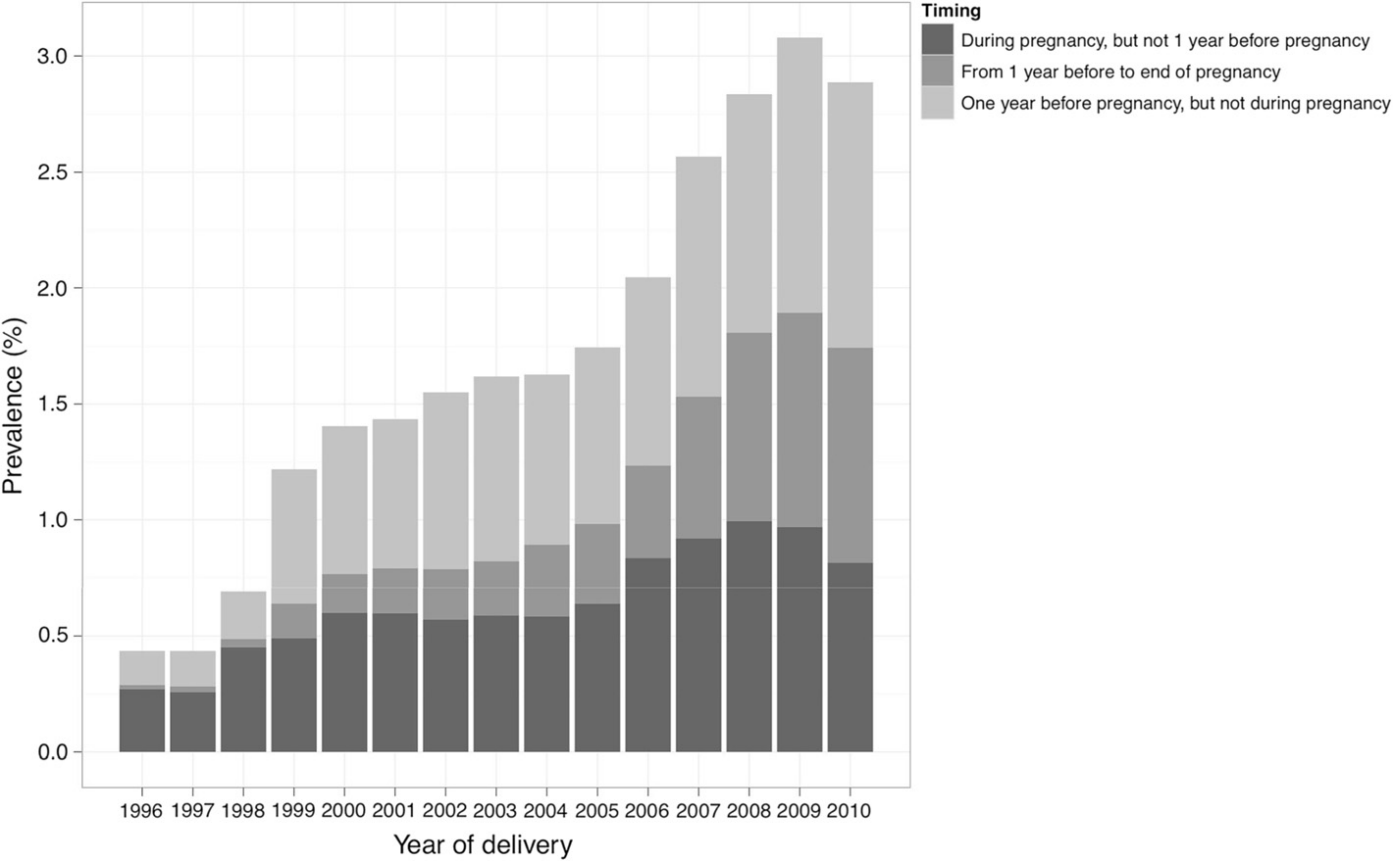
**The annual prevalence of diagnosed depression and depression-related psychiatric disorders from one year before or during pregnancy in women giving birth in Finland, years 1996–2010.** Data on deliveries are linked to both outpatient (1998–2010) and inpatient data (1996–2010) of the women.

The prevalence of diagnosed depression and depression-related psychiatric diagnoses (ICD-10 codes F20-F48) in the whole pregnant population within the period of one year before pregnancy until the end of pregnancy was 1.7% (years 1996–2010). Only inpatient data were available for years 1996–1997, accounting for the lower prevalence in these years’ cohorts (Figure 4). The cumulative incidence of any registered psychiatric or neurodevelopmental disorder (ICD-10 F00-99) in the study population including all live born singleton offspring born during 1996–2010 was 6.9% in 2010 (age range 0–14 years), and 12.9% in 2010 among the oldest offspring cohort (age 14, born in 1996).

## Discussion

The FinESSI study represents a population-based birth cohort study of SSRI use and infant/childhood outcomes until age 14, capitalizing on a unique and highly valuable sample consisting of all singleton offspring of women in Finland who received SSRI’s during pregnancy from 1996–2010. The data in all the included national registers have high quality [24,32], and the population-based setting allows for good generalization of the study results.

These resources provide the study with several design advantages, including minimization of ascertainment bias and bias due to loss of follow-up. Further, the registers allow accurate assessment of SSRI dosage, timing and duration of exposure. Based on the data obtained from the Medical Birth Register, we are able to adjust for several confounding influences and modifying factors related to pregnancy and perinatal conditions which may affect long-term neurodevelopment. By controlling for paternal and grandparental history of psychiatric illness and maternal psychiatric illness after the index pregnancy we can further adjust for potential genetic and environmental factors modifying outcome.

We observed a steadily increasing use of SSRIs in the Finnish pregnant population, from 0.4% in 1996 to 3.8% in 2010. This is in line with observations in other countries [1,33,34]. The prevalence of diagnosed maternal depression and depression-related psychiatric disorders was 1.7% in the whole pregnant population covering the period of one year before pregnancy until delivery, and increased during the study frame. Together with the increasing use of SSRIs, this probably indicates a growing awareness of the potential perinatal risks related to untreated prenatal depression [35].

Some limitations related to the methodology exist. Register-based studies may be biased by lack of drug compliance. Another limitation is that data on illicit drug and alcohol use are not routinely collected in the registers. Third, the study includes information on offspring who have been treated for psychiatric disorders, but lacks information on untreated cases. However, Finland has a health care system in which most services, including universal coverage of psychiatric and medical conditions, are provided and financed by the public sector (state and municipals). This increases the likelihood of getting treatment when treatment is sought, thereby increasing the likelihood of detecting these disorders in the registries. Hence, both severe and mild cases are also likely to be detected.

## Conclusions

This study will provide information on the possible long-term psychiatric and neurodevelopmental sequelae of prenatal exposure to SSRIs. The national registers offer an extremely valuable database for this purpose. Given the widespread use of antidepressants, and the considerable prevalence of depression and other psychiatric disorders during pregnancy, we expect that this research will have considerable implications for prescribing practices for clinicians. Through integration of this project in a large research collaboration encompassing many disciplines, this study serves as a model for translational epidemiology, maximising the strengths of experimental, epidemiologic and clinical studies [20].

## Abbreviations

ATC: Anatomic-Therapeutic-Chemical; DRR: Drug Reimbursement Register; HDR: Hospital Discharge Register; 5-HT: Serotonin; ICD: International Classification of Diseases; KELA: Social Insurance Institution in Finland; MBR: Medical Birth Register; PIC: Personal Identity Code; PR: Population Register; RCM: Register of Congenital Malformations; SSRI: Selective Serotonin Reuptake Inhibitor; THL: National Institute for Health and Welfare.

## Competing interests

The authors declare that they have no competing interests.

## Authors’ contributions

All authors have contributed to the manuscript in the conception and design, in drafting and revising the article critically, and approved of the final version.

## Source of funding

This study is funded by NIH Grant P50MH090966 (Project 1, A. Sourander and A. Brown, co-PIs) and the Sackler Center for Developmental Psychobiology at Columbia University.

## Pre-publication history

The pre-publication history for this paper can be accessed here:

http://www.biomedcentral.com/1471-244X/12/217/prepub
